# Challenges faced by rural people in mitigating the effects of climate change in the Mazungunye communal lands, Zimbabwe

**DOI:** 10.4102/jamba.v11i1.596

**Published:** 2019-02-21

**Authors:** Louis Nyahunda, Happy M. Tirivangasi

**Affiliations:** 1 Department of Social Work, University of Limpopo, South Africa; 2 Department of Sociology and Anthropology, University of Limpopo, South Africa

## Abstract

The phenomenon of climate change is one of the most contested and debated concepts globally. Some governments still deny the existence of climate change and its impact on rural–urban areas around the world. However, the effects of climate change have been visible in rural Zimbabwe, with some communities facing food insecurity, water scarcity and loss of livestock. Climate change has impacted negatively on agriculture, which is the main source of livelihood in Zimbabwe’s rural communities. This study aims at exploring challenges faced by rural people in mitigating the effects of climate change in the Mazungunye community, Masvingo Province, in Zimbabwe. The objectives of the study were to identify the challenges that impede effective adaptation of rural people to climate change hazards and to examine their perceptions on how to foster effective adaptation. The researchers conducted a qualitative research study guided by descriptive and exploratory research designs. Purposive sampling was employed to draw the population of the study. The population sample consisted of 26 research participants drawn from members of the community. Data was collected through in-depth individual interviews and focus group discussions. Thematic content analysis was used to analyse data. The findings of the study revealed the following challenges: unpredictability of indigenous knowledge systems, lack of resources and technoscience adaptive methods, lack of support to implement viable mitigation strategies, lack of information about resilience and adaptive capacity to climate change. This study has significance to policymakers and other stakeholders concerned with devising and implementing policies and programmes that are responsive to rural people’s needs in the climate change terrain, tapping into their presenting challenges as a departure point for intervention. The study recommended that the most important way to help rural poor people adapt to climate change is through the provision of information; immediate response to needs and climate-smart agricultural policies.

## Introduction and background

Climate change–related disasters have escalated around the world, causing a serious threat to the existence of humans and their livelihoods (Mugambiwa & Tirivangasi 2017; Mugambiwa 2018; Tirivangasi 2018). Climate change affects agriculture, depletes water resources and impacts on food security (Chikosi, Mugambiwa, Tirivangasi & Rankoana 2018). Climate change, which is attributable to the natural climate cycle and human activities, has adversely affected agricultural productivity in Africa (Mugambiwa & Tirivangasi 2017; Ziervogel et al. 2006). There is ample evidence that reveals that Africa and other developing countries face more challenges from climate change because of poor adaptation mechanisms in place (Enete & Achike 2008; Jagtap 2007; Nwafor 2007). Zimbabwe’s rural community, too, has been negatively affected by the impacts of climate change. This can be evidenced by the increase in unreliable rainfall patterns, resulting in nationwide flash floods and droughts. This has left communities that rely on farming outputs at greater risk, because climate change diminishes the prospects of having a great yield every year.

Ndebele and Mubaya (2015) highlighted that climate change introduces greater variability in maize yields, thus making maize production a riskier agricultural activity. According to Ndebele and Mubaya (2015), in Masvingo Province’s two districts of Bikita and Zaka, dry spells are on the increase; rainfall patterns have become more erratic, therefore failing to support crop production. Masvingo Province has a strong likelihood of becoming a non-maize-producing area because of climate change (Matarira, Makadho & Mukahanana-Sangarwe 2004). This calls for the need to improve communities’ food security by encouraging farmers to grow small-grain crops such as sorghum and millet, which are more tolerant to drought conditions than maize. However, the government has not done much to fund the growing of small-grain crops, which have been proved to be a viable means to mitigate the effects of climate change (Chazovachii, Chingwenya & Mushuku 2012). This can be cited as poor strategic planning on the part of the government, which keeps distributing maize seed in areas that are prone to drought instead of encouraging people in these areas to grow small-grain crops (Manyeruke, Hamauswa & Mhandara 2013).

Gukurume (2013) adds that what should be underscored is that the perpetual decline in rainfall and skyrocketing water shortages in the region pose serious implications for rain-fed agriculture, which predominates in Bikita District. Harvest failures are perpetual in most parts of Bikita. In their study in Zimbabwe, Matarira et al. (2004) established that yields of maize, the most widely grown crop in Zimbabwe, decreased dramatically under dry land conditions in some regions (sometimes up to 30%) even under full irrigation conditions because of high temperature increases that shorten the farming season. Chazovachii et al. (2012) argues that commercialisation of the maize crop in Zimbabwe has also forced farmers in marginal lands to grow maize even though their agro-ecological conditions are unsuitable for maize faming, which has resulted in severe shortages of food. Hence, there is a growing feeling that crop yield and staple food grain production in marginal areas have become highly variable because of this shift.

Further, Manyeruke et al. (2013) indicated that in 2012 the government of Zimbabwe declared potato as a strategic food security crop. However, much has been done to assist farmers with funds to intensify production of this crop. Many farmers have not taken up potato farming because it is a capital-intensive crop that requires huge initial capital investment. The government has also not done much to provide extension services to farmers who want to venture into potato farming. Zimbabwe still experiences and is compromised by the presence of various socio-economic stresses which may interact with climate change impacts to increase community vulnerability and reduce adaptive capacity (Conway 2009). The socio-economic stresses contribute to and compound the impacts of current climate change in Africa and will have negative effects on the country’s ability to cope with climate change (Conway 2009). Such stresses include rampant poverty; various political, ethnic and economic conflicts; ignorance; lack of skills; low level of technological advancement; weak institutional capacity; limited infrastructure; lack of technology; lack of information and poor access to resources by the majority (Lisk 2009).

Ndaki (2014) observes that even though education is prioritised on the climate change agenda, the knowledge level regarding climate change adaptation in some of the regions remains low. In addition, conceptualisation of key issues like climate change adaptation, capacity to adapt to climate change and vulnerability level to climate change require highly elaborate work to reflect the local contexts of an exposure unit or system in question (Below et al. 2012). Except for natural adaptation which normally takes place in natural systems, most adaptation actions require decision-making. However, the extent to which decisions are well-informed and rational remains questionable. This is because, much as scientific projections help to provide some view of what will happen in future, many uncertainties in climate change knowledge exist because of the inability of human beings to be exactly sure of what will happen in the climate, human and social systems as well as the specific time frame, for example, the next 50–100 years (Tompkins & Adger 2004). This shows the magnitude of challenges facing rural communities, where information access is limited.

The international community has also not been successful in addressing climate change impact; hence, industrial emissions are still on the increase despite debates and emission targets in the Kyoto Protocol (World Meteorological Organization 2012, 2013). This means that as emissions increase, the impacts and their severity will also increase in future, not allowing an opportunity for successful adaptation and resilience building for developing countries. Ndebele and Mubaya (2015) point out that among the cited barriers to efforts to mitigate the negative effects of climate change in Zimbabwe, Ethiopia and South Africa are lack of access to credit, fertile land, extension services and climate change information, including a limited concrete institutional response among government institutions nationally and between different levels of government with communities.

### Problem statement

Climate change as a fact of life is particularly formidable to low income rural communities whose livelihoods heavily depend on rain-fed subsistence agriculture like those in the focus of this study, the Mazungunye communal lands (IPCC 2012). According to Hellmuth et al. (2007), climate change presents risks to lives and livelihoods at the individual level and to the economy and the infrastructure at regional and national levels. Rural people are believed to be particularly vulnerable to climate change. Their vulnerability is not attributed to climate change only but is also a combination of social, economic and environmental factors that interact with it (Turpie & Visser 2013). A study conducted by Chazovachii et al. (2012 concluded that communal farmers in Masvingo Province have not been passive victims of the vagaries of climate change and variability. They have rationally responded to it through various adaptation and mitigation strategies, both individually and collectively. However, as observed by Ofoegbu et al. (2015), rural communities have remained vulnerable to climatic-induced shocks although they are employing a plethora of mechanisms to mitigate the effects of climate change. This is because their high exposure to climate change risks does not match their adaptive capacity. The researchers identified such factors (challenges) as barriers to adaptation, which became the focal point of the study. According to Gukurume (2014), in Zimbabwe many studies have tried to understand the effects of climate change on agriculture, health and the economy, as well as strategies to mitigate climate change, just to mention a few, but there is little evidence that any studies have been dedicated to unearthing the challenges faced by rural people in their efforts to mitigate the effects of climate change specifically in the Mazungunye communal lands. Several studies on climate change have focused on industrialised countries or urban areas, thereby ignoring rural communities in the developing world that are engulfed in more complex realities of the climate change phenomenon (Mahiya & Gukurume 2012). It was the fundamental intention of this study to close the knowledge gap by unravelling the challenges faced by rural people in mitigating the effects of climate change in the Mazungunye communal lands.

### Climate change adaptation in rural Zimbabwe

In Zimbabwe, as elsewhere in Africa, most rural households depend on natural resources for subsistence and livelihoods (Dube & Phiri 2013; Gukurume 2012; Ndebele & Mubaya 2015). The vulnerability of rural households to climate risk may be linked closely to socio-economic conditions, which correlate with the people’s adaptive capacity (Ofoegbu et al. 2015). Most rural communities in Zimbabwe are underdeveloped infrastructurally and the dependence on climatic volatile resources is very high. Their livelihood activities are reliant on the natural environment, savaged by climate change, which plunges these communities into climate change vulnerability (Dube & Phiri 2013). Gukurume (2014) observed that climate change has aggravated poverty levels in most rural communities of Zimbabwe, where 70% of the country’s population is trapped in high poverty circles and mostly located in rural areas. According to Chagutah (2010), Zimbabwe is particularly vulnerable because of its heavy dependence on rain-fed agriculture and climate-sensitive resources. Climate change variability has intensified problems as evidenced by declining agricultural outputs, decline in economic productivity, poverty and food insecurity, with rural people mainly affected. Mugabe and Chimutambgi (2016) indicated that the government of Zimbabwe declared drought a national disaster on 05 February 2016 because of the dire effects of the El Niño weather. The declaration was meant to ensure a well-coordinated response to minimise distress and suffering caused by the climate change variability mostly in Masvingo and Matabeleland provinces, which are seriously engulfed in the catastrophic effects of climate change. These provinces are naturally situated in a region that receives low rainfall.

Rural people are engaging in a plethora of activities as resilience mechanisms to withstand the effects posed by climate change. However, some of their income-generating activities are reliant on agricultural and forest products, which are susceptible to climate change and variability. This is perpetuating the poverty levels in Zimbabwe’s rural communities because rural people are executing mechanisms that are not responsive to the changing climate (Madzwamuse 2010). Adaptive capacity among rural people is typically limited by poverty, poor public and environmental health, weak institutions, lack of infrastructure and services, marginalisation from decision-making processes and planning procedures, gender inequality, lack of education and information, natural disasters, environmental degradation, reliance on rain-fed agriculture and climate-sensitive resources, and insecure tenure (UNFCCC 2014). According to the International Fund for Agricultural Development (2015), poor rural households are highly exposed to shocks because their livelihoods depend on an increasingly deteriorating natural resource base and on often-volatile climatic conditions. They are also particularly vulnerable to shocks because they have few assets to fall back on and limited risk management strategies. Yohe and Tol (2002) noted that adaptive capacity has its own determinants, and in any system it can be prudent to assert that individuals, communities and institutions have such determinants: (1) the availability of technological advancements that enable adaptation, (2) the equal distribution of resources, (3) availability of human and social capital including literacy levels, social and personal security and (4) how the public perceives the cause of disturbances (climate change) and its manifestations. The researchers affirmed that the envisaged determinants of adaptive capacity are still far from being a lived reality in Zimbabwe’s rural communities, where poverty is at the centre stage of people’s livelihoods, aggravated by climate change.

## Theoretical framework

This study was guided by resilience theory as an overarching framework. According to Daniel (2011), resilience theory was coined by Holling in 1973; he denoted that the theory determines the persistence of relationships within a system and is a measure of the ability of these systems to absorb changes of state, driving variables and parameters and to persist. Since then, the concept has gained traction and spread into many research disciplines including engineering, psychology, philosophy, economics and so forth (Folke 2006). Resilience theory emphasises strengths over problems and incorporates key contextual factors in its structure. It emphasises transactions between the developing person and the social and physical environments. It is defined as the process of, capacity for or outcome of successful adaptation despite challenging or threatening circumstances (Masten, Best & Garmezy 1990). The concept of resilience provides a useful lens for examining the challenges faced by rural communities in mitigating the effects of climate change in Zimbabwe.

The term ‘resilience’ has been used to label three aspects: (1) individuals who have experienced traumatic events but have been able to recover well; (2) persons who belong to high-risk groups but who have more favourable outcomes than expected and (3) persons who show positive adaptation despite life stressors (Masten et al. 1990). In applying resilience theory in the current study, the researchers could identify the vulnerability of the Mazungunye communal lands and assess whether the community members were in a position to be able to recover from the effects of climate change. The Mazungunye communal lands as a rural community is identified as one of the communities affected by climate change-related disasters in Zimbabwe, hence the need to concentrate this research in this area. Resilience has many forms that range from individual, family and social resilience to ecological resilience (Pimms 1984).

According to Gunderson and Holling (2002), people’s resilience or capacity to manage and adapt to change is determined by their assets, including the amount and quality of knowledge and labour, physical and financial capital, social relations and networks. Further, this includes services they can access such as transport and communication, access to credit, markets, and emergency relief and recovery systems. The study operated within the parameters of resilience theory, which was a navigating tool that assisted the researchers to gather in-depth information about the resilience strategies of the Mazungunye communal lands, including the coping and adaptive mechanisms that they employ in the face of climate change. This included understanding the shocks and stresses that affect the community and its systems and the factors that render the community vulnerable to those shocks and stresses, particularly those related to climate change.

### Study area

**FIGURE 1:**
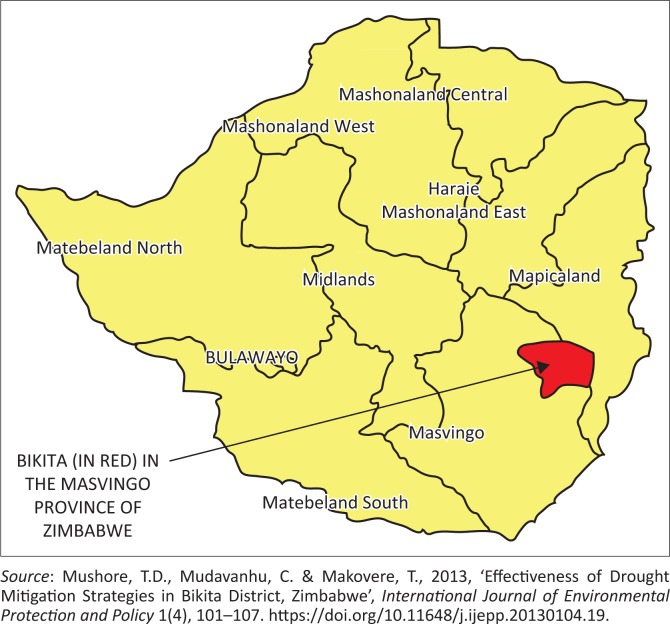
Map of Zimbabwe showing Bikita District (indicated in red) where the Mazungunye community is located.

**FIGURE 2:**
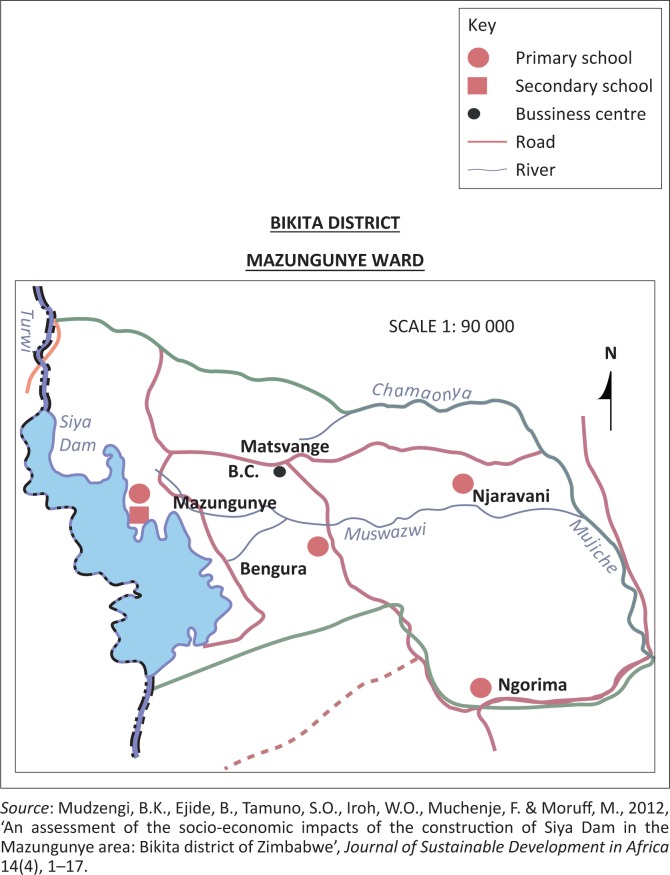
The location of Mazungunye Ward.

The Mazungunye communal lands are found in Bikita District of Masvingo Province, Zimbabwe. Masvingo Province is in the south-eastern part of Zimbabwe. It borders Mozambique on the east, South Africa to the south, Matabeleland South Province to the west, Midlands Province to the north-west and Manicaland Province to the north-east. According to Simba, Chikodzi and Murwendo (2012), Bikita is approximately 80 km east of Masvingo Urban. Bikita covers an area of 10 000 square kilometres and has a population of 20 000. Unganai and Murwira (2010) note that the district is susceptible to seasonal droughts, the 1992, 2002 and 2008 droughts being the most notable events. Mazungunye lies in Zimbabwean Agro-ecological Region 4. This is a semi-intensive farming region experiencing a mean annual rainfall between 300 and 600 mm with a 40% – 45% coefficient of variation. It is subject to periodic seasonal droughts and prolonged dry spells during the rainy season (Mudzengi et al. 2012). Mean annual temperature is between 25.0 °C and 27.5 °C (Chenje, Sola & Paleczny 1998). The study area is made up of six villages: Bengura, Marufu, Jere, Ngorima, Njaravani and Chiwawa. The majority of people in the study area are peasant farmers practising farming based on growing crops and livestock rearing. The crops grown include maize, groundnuts, roundnuts, rapoko and sorghum (Mudzengi et al. 2012). The Mazungunye Ward in Bikita District is shown in Figure 1 and 2.

## Research methodology

### Research designs

The study was guided by descriptive and exploratory research designs. Kothari (2005) posits that descriptive research seeks to gather fertile data and in-depth understanding of the phenomenon under study. In this case the study sought to understand the challenges faced by people in the Mazungunye communal lands in their efforts to mitigate the impact of climate change. This research design was chosen as it is renowned for producing quality data and it is in-depth in nature (Creswell 2007). The researcher made use of the exploratory research design as well. Creswell (2007) indicates that the main aim of exploratory research is to identify the boundaries of the environment in which the problems, opportunities or situations of interest are likely to reside and to identify the salient factors or variables that might be found there and be of relevance to the research. He adds that this is the most useful research design for those projects that are addressing a subject about which there are high levels of uncertainty and ignorance and when the problem is not very well understood. The exploratory design was suitable for this study, which was conducted in a marginalised rural community.

### Population and sample size

The sample size of this study was drawn from the Mazungunye community in Masvingo Province in Zimbabwe. The purposive sampling method was followed by the researcher. Dawson and Catherine (2002) state that in purposive sampling, the sample is selected based on the researcher’s knowledge of the population, its elements and the nature of research aims. The population sample consisted of 21 research participants drawn from members of the community, two village heads and two Non-Governmental Officials as shown in Table 1.

**TABLE 1:** The respondents to interviews and focus group discussions

Targeted respondents	Targeted number	Actual number
Ordinary villagers	24	21
Village heads	2	2
Non-governmental organisation officials	3	3

**Total**	**29**	**26**

### Data collection and analysis

The researcher collected data using unstructured in-depth interviews and focus group discussions. The data collection process was more interactive and this allowed the participants to open up during the data collection process. Focus groups were comprised of individuals selected and brought together in a group to discuss their views on climate change and the challenges they face in their climate change adaptation efforts.

Individual interviews were used with village leaders and Non-Governmental Organisations (NGOs) officials, whereas focus group discussions were used with community members. The researchers conducted three focus group discussion sessions with members of the community. Each group had eight participants. The Shona language was used, as the dominant language in which the participants could easily articulate their views. The researcher made use of a digital audio recorder during data collection to ensure that important information would not be lost in the process. The respondents who took part in the focus group discussions were aged 40 and above, and all of the community members had been in the community for the previous 10 years. This group was believed to possess rich knowledge about climate change in the area. The total number of women who took part in the study was 16 as compared to 10 for men. It was established that the reason behind the high turnout of women is that they were easily accessible and more accommodating, while men were hard to reach, often citing other commitments.

The researchers used thematic analysis for the analysis of data. According to Guest, Namey and Mitchell (2012), thematic analysis is the most common form of analysis in qualitative research, which emphasises pinpointing, examining and recording patterns or themes within data. The analysis involves six phases, which begin with becoming familiar with the data, generating initial codes, searching for themes, reviewing themes, defining and naming themes and finally producing the report. In this study the researchers immersed themselves in the collected data and became familiar with it through careful reading and rereading of the data (Rice & Ezzy 1999). The interview guides and transcripts were translated into English and back to Shona to ensure that the meanings were not lost in the process. The researchers had to find English meanings for some of the information submitted by the respondents and where no corresponding meanings in English were available, Shona words were used. The researchers then created an overall narrative with all of the data and analysed each theme and its individual narrative, whether or not any of the themes contained subthemes. The last stage involved the final analysis and write-up of the report, whereby the researchers provided sufficient evidence of each theme using vivid examples from the collected data.

## Results and discussion

This section presents and discusses the results of the study, in the form of the challenges faced by rural people in mitigating the effects of climate change in Zimbabwe. The Mazungunye community was hit hard by the adverse effects of climate change, like many other rural communities in Zimbabwe. The inhabitants of the community are doing what it takes to withstand the adverse effects of climate change, but in the process their efforts are being crippled by several challenges. From the information gathered through interviews with NGO officials and village leaders as well as the focus group discussions with community members, several challenges were cited by the respondents and are discussed in this section under the subsequent themes.

### Subtheme 1: Unpredictability of indigenous knowledge systems

There was a general view among the respondents that indigenous knowledge systems (IKS) used to play a critical role in predicting weather events for the people of the Mazungunye community; however, this has changed mainly because of climate change and thus the people no longer have faith in them anymore. One of the village leaders echoed the following:

‘We used to rely more on indigenous knowledge for productive farming in our community. Heavy rains were mostly characterised by budding of new leaves on a dry trees, rumbling sounds from hills or flames on mountains; the belief was heavy rains would put out the fire. Low rainfall is predicted by the presence of birds’ nests, which they build on lower levels of the trees.’ (Village leader, Male, 76 years of age)

The same was submitted in the focus group discussions, that there is a lot of mystery surrounding the reliability of the long-trusted IKS for predicting weather patterns. Therefore, the changes in the IKS are a cause of concern; as they are no longer reliable, the villagers can no longer predict what is ahead of them. Masendeke (2008) submitted that although people in rural areas in some instances concede that they can no longer rely solely on their traditional knowledge, indigenous knowledge relating to climate change, whether it concerns agricultural techniques, biodiversity, indicators of change or weather prediction and response, provides the basis for many successful and cost-effective adaptation measures. Indigenous knowledge transmission is threatened by social, cultural and environmental drivers, including climate change, resulting in erosion of the knowledge base and its potential to respond to climate change (UNESCO 2013). According to the sentiments submitted by the respondents, they are saddled with confusion as to which knowledge is viable because the predictions from the Department of Metrological Services are not reliable as they contrast with the change in climate. For the Mazungunye community, which has a long history of reliance on IKS, its unpredictability is posing a serious threat to its reliability, thereby making the mitigation of the effects of climate a big mountain to climb.

### Subtheme 2: Lack of resources and technoscience adaptive methods

This study found that one of the major challenges affecting the efforts of the Mazungunye community to mitigate climate change is lack of technoscience adaptation methods, which include drilling of boreholes, dam construction for irrigation, supplementary feeding and reliance on food aid. The community is located at a walkable distance from Siya Dam, one of the biggest dams in the district, which borders Bikita and Zaka Districts. The dam supplies irrigation services to the people in Zaka District at Kangera. However, the dam is drying up because of ongoing heat waves as a result of climate change. The respondents in all focus group discussions expressed disappointment towards the government’s stance in ensuring that the dam benefits them for agricultural purposes. This is what one of the respondents had to say:

‘You see that dam was supposed to be our safety net, but that is not the case. Now it has dried up and we can safely say we never benefited from it. We have received endless and futile promises from the government about constructing irrigation systems from Siya Dam but more than 30 years have lapsed now and nothing has materialised. I don’t think we would be suffering like this if we had irrigation facilities. At one point in time there were government officials who came and put some pegs earmarked for irrigation installation outside Mazungunye High School premises as an appropriate place for irrigation but it just ended there. It’s very disappointing that Siya Dam only benefits people in Zaka.’ (Male, 68, community member)

The Mazungunye community is heavily dependent on rain-fed agriculture, which continues to deteriorate year by year, and this is making it very difficult for the community members to mitigate the effects of climate change. Further, 2020 rain-fed agricultural yields are most likely to decrease by 50% in other African countries (IPCC 2007). According to Dube and Phiri (2013), facilitation of drought preparedness and mitigation through appropriate technologies, including use of remote sensing, local weather forecasting, drought-tolerant crops, early warning information systems, irrigation technology and the building of resilience in rural communities are essential strategies.

Additionally, the study findings revealed that the level of development in the community is stagnant inasmuch as determinants of technoscience adaptive methods are concerned. Drinking water is a serious problem in the community – there is only one borehole for 240 households. Sometimes the borehole dries up and people are forced to walk for 7 km to fetch water from other communities. It was submitted by the respondents that the borehole was drilled in 1986 by a certain donor from Caritas Internationalis which is a Roman Catholic relief agency. Intergovernmental Panel on Climate Change (IPCC) (2007) noted that for there to be effective adaptation to climate change, there is need for rainwater harvesting expansion, expansion of water reservoirs that store water, irrigation competence and water use efficiency as well as desalinisation. Rural communities in Zimbabwe are poorly resourced in terms of technology and rely heavily on basic tools; their agricultural capital is limited (Mutekwa 2009).

The researchers argue that lack of ethnoscience methods in the community is making mitigation of the effects of climate change more complex. On the same note the government policy objectives should focus on water availability through the expansion of irrigation for the small-holder sector, water harnessing through construction of dams and other methods such as water as water-harvesting techniques and equitable allocation and efficient use of scarce water. Section 3 of the Zimbabwe Climate Change Response Strategy of 2012 puts emphasis on strategy enablers such as capacity building; technology transfer; climate change education, communication and awareness (United Nations Framework Convention on Climate Change 2012).

This study established that the inhabitants of the community are plunged in abject poverty, which has been crippling them for years, and climate change is adding an unbearable burden on them inasmuch as they are crawling to withstand its effects. The community is very marginalised and underdeveloped, where most respondents submitted that they were living from hand to mouth. Poverty is making climate mitigation difficult for the Mazungunye community. Officials from NGOs were of the view that the departure point to address the effects of climate change in the community is to address the level of poverty in the community.

This is supported by Brooks and Loevinson (2011), who indicated that one of the most important ways to help rural poor people adapt to climate change is to address rural poverty. Government policy actions will help increase poor people’s resilience to climate change if they achieve broad-based economic growth that reaches the poor; improve productivity in crops that are important to poor farmers and consumers; and strengthen trade to cope with subregional disparities in the agricultural effects of climate change. Ndaki (2014) is of the view that financing climate change adaptation remains a major challenge for developing countries, whose economies and livelihoods are so vulnerable to climate change impacts. This is the case in Zimbabwe, where it is evident that there is poor policy implementation to curb the effects of climate change; although the Zimbabwe Climate Change Response Strategy is in place, it has not yielded the desired results when looking at the fragile livelihood circumstances in the Mazungunye community.

### Subtheme 3: Lack of support to implement viable mitigation strategies

The respondents expressed disappointment at the government’s reluctance to assist people with their food challenges. According to Easton and Sommers (2003), the government is expected to carry out programmes that incorporate people’s ideas in order to come up with policies that address the needs of the people. The respondents noted that the government used to provide subsidised maize as a climate change mitigation strategy through the Grain Marketing Board (GMB), which the people would purchase at Nyika Growth Point; the programme ran and ended in 2011. Furthermore, the respondents expressed that such an initiative was very good for them as it increased their access to food. However, the respondents noted that some of the maize did not reach the people in need but was purchased at a large scale by the government. Responses from focus group discussions and interviews with village leaders showed that Mazungunye is ensnared in a serious food crisis and all the people’s efforts are coming up short because the government is very passive in terms of intervention. However, the respondents opined that this is not surprising, meaning that it is the usual scenario that the government does not do much to help people in the area. This displays a lack of responsiveness on the part of the government.

This study ascertained from the respondents that the food crisis in the community is serious as there was no maize at the Grain Marketing Board (GMB), and that maize sold by individuals was offered at an exorbitant price of between $8.00 and $10.00 per 20 kg bucket. The respondents further pointed out that they exchanged their cattle for bags of maize, with one beast going for as little as six bags of maize. As Davidson et al. (2003) observed, the situation reverses the developmental prospects of the respondents. This in turn impoverishes the people in the community, as they end up with nothing, which affects all the efforts towards mitigating the effects of climate change. The respondents appreciated the government’s efforts in introducing the Operation Maguta project in 2007, where the government of Zimbabwe provided farming inputs such as fertilisers, seeds and tractors. However, there were mixed reactions from the respondents as to whether the project achieved its targeted objectives. Some respondents felt that only a few people benefited from the programme, and they viewed beneficiation as being based on one’s political affiliation. There was a consensus among many respondents that the programme died a natural death. This is what came out in one of the focus group discussions:

‘The government tried its utmost best to mitigate the effects of climate change through the Operation Maguta programme, but the scheme died a natural death because of corrupt officials. In this community, only a few people benefited from the scheme. Some of the inputs and equipment picked up dust and rust without even reaching us. The main focus was on resettled farmers and little consideration was extended to peasant farmers like us. We witnessed the highest level of corruption in distributing such equipment. Now the government is passive in terms of distributing small-grain seeds. They keep on distributing maize seeds, which have proved to be a failure in this community.’ (Community member, Female, 52 years of age)

Manyeruke and Hamauswa (2012) point out that Zimbabwe has been held back by corruption, debt crisis and non-transparency as well as conflicts. According to the respondents, there is not much diversification from maize, inasmuch as they are striving to grow small-grain crops. Chazovachii et al. (2012) noted that the government of Zimbabwe has not done much to fund the growing of small-grain crops, which has been proven to be a viable means to mitigate the effects of climate change.

This can be cited as poor strategic planning on the part of the government, as it keeps distributing maize seed in areas that are prone to drought instead of encouraging people in these areas to grow small-grain crops (Manyeruke et al. 2013). Zimbabwe’s government, therefore, as a third world country has weak inter- and intrasectoral coordination insofar as climate change is concerned. Therefore, the country has a narrow capacity for climate change policy analysis, implementation and limited resources to fund climate change adaptation and mitigation programmes (Ndebele & Mubaya 2015). The key areas that the respondents cited as lacking support include food security, irrigation-supported agriculture, improvement of water sources, improved distribution of small-grain crops and climate change education.

### Subtheme 4: Lack of information about resilience and adaptive capacity to climate change

The respondents lamented that they had not received information about resilience and adaptive capacity to climate change, amidst assertions by officials from NGOs that they were trying their level best to educate the community on climate change resilience and adaptive capacity. In all the focus group discussions, the researchers noted that there was a high level of appreciation for the role being played by NGOs operating in the Mazungunye community, though the NGOs’ special focus was on the provision of material support to help in mitigating the effects of climate change. In one of the focus group sessions, the following was said:

‘We appreciate the role that NGOs such as Cooperative for Assistance and Relief Everywhere (CARE), GOAL and Action Faim are playing in meeting our needs in times of drought resulting from climate change like this. The organisations are supporting us in the best way they can, but we haven’t learned much about the causes of this drought, and it is their role to educate us even on how best to remain strong.’ (Non-Governmental Organisation official, Male, 45 years of age)

On the other side, an official from Action contre la Faim maintained that they in some instances tried to educate the community about climate change and resilience, but most of the workshops that they tried to convene faced poor attendance. It was further suggested that the community had developed a high level of dependency syndrome, where each time a community meeting was organised by officials from NGOs, people would be expecting to receive material things, not just information. As has been mentioned before, the community is unconsciously making ends meet in terms of employing a number of mechanisms to mitigate the effects of climate change. They are just unaware that they are being resilient. The other paradox has to do with the misconception about drought, which the communities view as climate change itself and not as an effect of climate change. It can be argued that the community’s knowledge base on climate change, resilience and adaptive capacity is very low and needs to be scaled up as the current status is affecting efforts towards mitigating the effects of climate change.

This finding is corroborated by Ndaki (2014), who contends that knowledge level regarding climate change adaptation in some regions remains low. Additionally, the role of information and skills in explaining adaptive capacity cannot be overemphasised. For a population to take initiatives to adapt to climate change, it has to be aware of the risks posed and perceive that something is not right and that there is a need for change so as to be able to adapt. For this to happen, people must have access to information. Access to information can help people assess the magnitude of the climate change challenge, possible options and those feasible within the relevant context (Tol 2009). It is the researchers’ view that the level of access to information with regard to climate change and best coping mechanisms needs to be heightened in the Mazungunye community to raise awareness that will in turn make adaptation to climate change easy.

## Conclusion

This study has revealed several challenges faced by the Mazungunye communal lands. The literature and the findings show that there is a need to depart from reliance on rain-fed food production through heavy utilisation of irrigation. The study established that rural people are enmeshed in a vicious circle of reinforcing traps of climate change and unfruitful efforts to escape its impacts. The government is not mainstreaming climate change into its rural development policies to the optimum, which is reinforcing stagnation in terms of development and execution of viable climate change responsive strategies for rural people in the Mazungunye communal lands. Additionally, the study revealed that early warning and response strategies are not being enhanced. The community members should be assisted by information or predictions of rainfall instead of relying on IKS information in terms of yearly rainfall patterns. The paper also argued that limited access to information is consistently noted as an impediment to building the much-needed adaptive capacity in Africa, and therefore information is an important resource in the fight against the adverse impacts of climate change. There is a need to give farmers regular information on current issues related to climate change and agriculture. This can be achieved through the strengthening of the nation’s extension services by involving local councils and local councillors. These are people close to farmers and they encourage farmers to form groups for enhanced capacity through group efforts. This may help them take advantage of the Internet.
